# Can Blood Biomarkers Help Predicting Outcome in Transcatheter Aortic Valve Implantation?

**DOI:** 10.3389/fcvm.2018.00031

**Published:** 2018-03-28

**Authors:** Cécile Oury, Alain Nchimi, Patrizio Lancellotti, Jutta Bergler-Klein

**Affiliations:** ^1^Department of Cardiology, Heart Valve Clinic, University of Liège Hospital, GIGA Cardiovascular Sciences, CHU Sart Tilman, Liège, Belgium; ^2^Gruppo Villa Maria Care and Research, Anthea Hospital, Bari, Italy; ^3^Department of Cardiology, Medical University of Vienna, Vienna, Austria

**Keywords:** TAVI, blood biomarkers, inflammation, myocardial stress, platelet, thrombocytopenia

## Abstract

Transcatheter aortic valve implantation (TAVI) has become the method of choice for patients with severe aortic valve stenosis, who are ineligible or at high risk for surgery. In this high risk patient population, early and late mortality and rehospitalization rates after TAVI are still relatively high. In spite of recent improvements in procedural TAVI, and establishment of risk models for poor outcome, determining individual risk remains challenging. In this context, current data from several small studies strongly suggest that blood biomarkers of myocardial injury, cardiac mechanical stretch, inflammation, and hemostasis imbalance might play an important role by providing informations on patient risk at baseline, and postprocedural progression of patient clinical conditions from days up to years post-TAVI. Although the role of biomarkers for predicting survival post-TAVI remains to be validated in large randomized studies, implementing biomarkers in clinical practice might improve risk stratification, thereby further reducing TAVI-associated morbidity and mortality.

## Introduction

Transcatheter aortic valve implantation (TAVI) has changed dramatically the treatment of severe aortic stenosis in inoperable patients or in patients at high risk for surgery. In the high risk population, particularly in the elderly, TAVI can offer a marked change in the life expectancy and quality of life of patients, and even nonagenarian patients can have successful valve replacement with acceptable periprocedural morbidity and mortality rates (1). However, early and late mortality after TAVI still remains relatively high. Results from registries and from the PARTNER trials reported 1 year all-cause mortalities between 22 and 30% (2–4). In order to improve patient evaluation and minimize futility, risk models for poor outcomes post-TAVI have been built and validated, providing Heart teams with important decision-making tools and informations (5–8). Since the prognosis of patients who benefit the most from TAVI is often not only determined by severe symptomatic aortic stenosis (AS), but also by multiple comorbidities, it would still be very useful to have parameters or biomarkers that would help to better predict the risk of major cardiovascular events for these patients.

**Figure 1:**
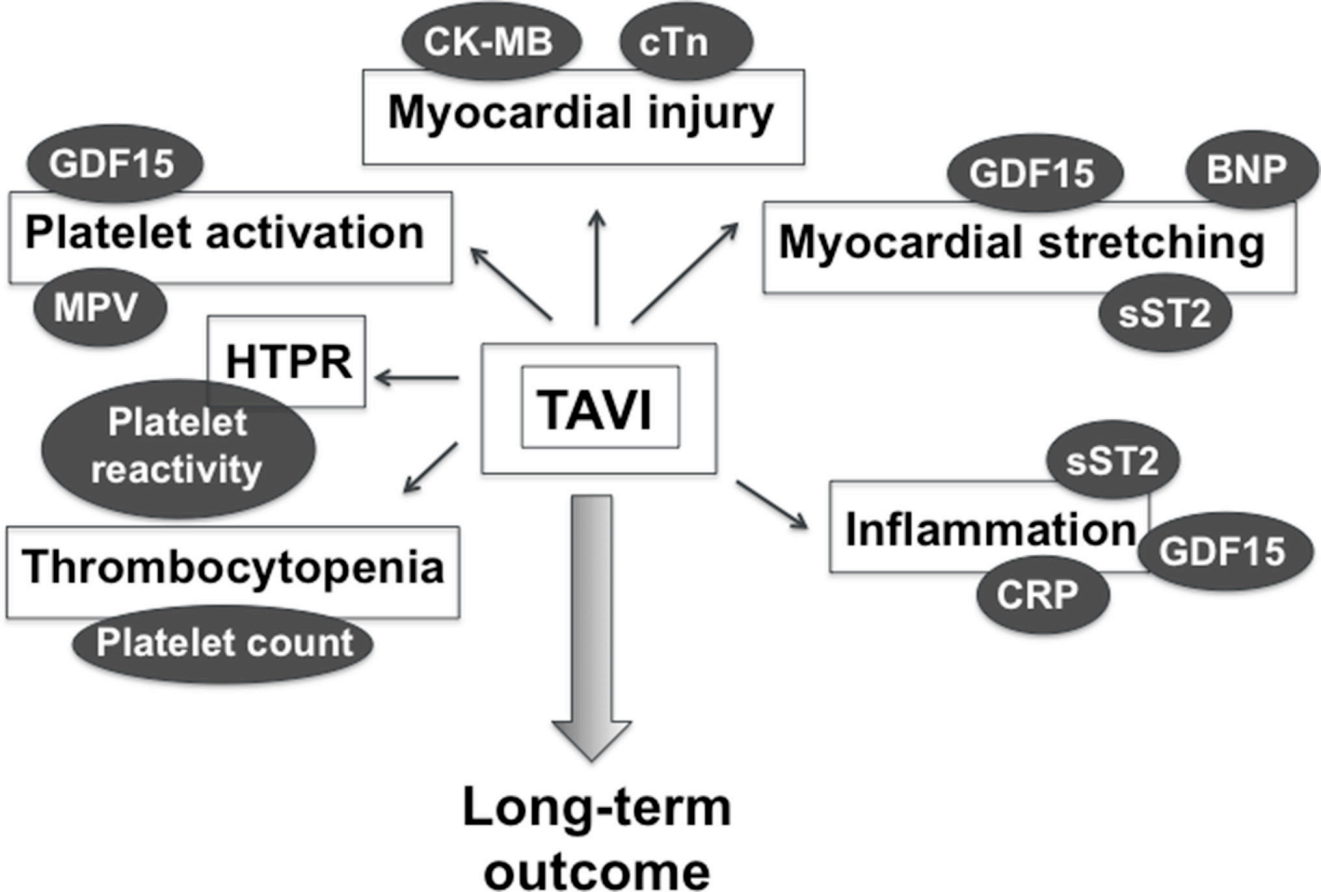
Blood biomarkers of TAVI-related myocardial injury, myocardial stretching, inflammation, and hemostasis imbalance that might provide postprocedural prognostic information. BNP, brain natriuretic peptide; CK-MB, creatinine kinase myocardial band; CRP, C-reactive protein; cTn, cardiac troponin; GDF-15, growth differentiation factor-15; HTPR, high on-treatment platelet reactivity; MPV, mean platelet volume.

Here, we present an overview of the role of most studied blood biomarkers for predicting poor outcome post-TAVI (Figure 1). Despite recent procedural advances that improved safety and flexibility of TAVI, these studies strongly suggest that biomarkers, in addition to risk scores, might help reducing further TAVI-associated morbidity and mortality, in a more personnalized manner.

### Markers of Myocardial Injury: Creatine Kinase Myocardial Band, Cardiac Troponin

Periprocedural elevation of cardiac biomarkers of myocardial injury is common in TAVI, with greater values observed following transapical or transaortic approaches compared to transfemoral (TF) approach (9). Higher levels of myocardial injury have been associated with reduced early and midterm survival following uncomplicated TAVI (10–13). Transapical (TA) procedure significantly associates with left ventricular apical fibrosis, contributing to apical wall motion abnormalities, which may, in turn, impair myocardial recovery (14).

TAVI clinical endpoints have been revisited in the current Valve Academic Research Consortium (VARC) −2 document (15), defining specific biomarker cut-off values for clinically significant myocardial infarction post-TAVI. In a large multicenter study of patients undergoing TAVI with different valve types and approaches, myocardial injury, determined by postprocedural rise in levels of creatine kinase myocardial band (CK-MB), was detected in two-third of patients undergoing TAVI, especially through transapical approach (16). Higher peak of CK-MB post-TAVI translated into impaired systolic left ventricle function at 6 to 12 months follow-up, and were associated with greater acute and late mortality (Table 1). Regarding cardiac troponin (cTn), correlation with patient outcome is less clear. Two small prospective studies of TF TAVI patients showed that baseline high sensitive TnT (hs-TnT) independently predicted survival in symptomatic high-risk patients with severe AS (18, 22). Post-procedural hs-TnT rose significantly after TF TAVI until day 3, which had prognostic value for 1 year mortality. Determinants of post-procedural hs-TnT were baseline renal function, duration of intraprocedural rapid spacing, as well as pre-TAVI hs-TnT values (18). Despite hemodynamic relief, cTnT levels did not normalize even after months following successful TAVI, suggesting that the prognostic value of cTn for 1 year patient outcome may rely on long-term changes in myocardial texture. A larger study indicated that cTnT elevation above VARC-2 cut-off within 12 h post-procedure was a strong independent predictor of 30 day mortality, and remained significant at 2 years (17). In disagreement with these findings, a more recent study indicated that, in contrast to CK-MB, cTn elevation above normal limit defined by VARC-2 had no impact on late mortality of patients undergoing TF TAVI (23). Notably, VARC-2 cTnI cut-off values failed to distinguish myocardial injury from type 1 myocardial infarction (angiographically high-grade coronary artery stenoses or occlusions) in TF and TA TAVI patients, and therefore could not be used as a marker of periprocedural MI (24). Furthermore, different cut-offs may apply to TA and TF patients. These results should still be confirmed in larger randomized studies.

**Table 1:** Proposed cut-off values of post-procedural biomarkers to predict mortality in TAVI.

Biomarker	Cut-off	Effect	References
CK-MB	>UNL (within 3 days post-TAVI)	↑30 day and late mortality in overall and non-TA TAVI	(11, 16)
	>5 × UNL* (within 3 days post-TAVI)	↑30 day and late mortality in overall and non-TA TAVI	
cTn	>15 × UNL* (within 12 h post-TAVI)	↑30 day and 2 year mortality (overall TAVI)	(17)
	≥166 pg/ml (3 days post-TAVI)	↑1 year mortality (TF)	(18)
BNP	Rise at 30 days post-TAVI	↑1 year mortality in TF TAVI	(19)
	>328 pg/ml (30 days post-TAVI)	↑1 year mortality in TF and transaxillary TAVI	(20)
	≥591 pg/ml (persistent from baseline to discharge)	↑2 year mortality in overall TAVI	(21)

UNL = upper normal limit based on the 99^th^ percentile values in a healthy population *according to VARC-2

BNP, brain natriuretic peptide; CK-MB, creatine kinase-myocardial band; cTn, cardiac troponin; TA, transapical; TAVI, transcatheter aortic valve implantation; TF, transfemoral.

### Markers of Myocardial Stretching: B-Type Natriuretic Peptides

Elevation of circulating B-type natriuretic peptides (BNP) that results from left ventricle myocardial stretching is commonly used in clinics to predict the onset of symptoms and adverse events in patients with severe AS (25–27).

Several studies performed on TAVI patients have assessed the value of preprocedural or serial BNP or of its biologically inactive N-terminal-proBNP (NT-proBNP) as predictors of postprocedural outcome. Initial studies found no association of baseline BNP or NT-proBNP levels and 2 month mortality after TF or TA TAVI (28, 29). A high BNP level in high-risk patients with severe AS was not an independent marker for higher mortality. These two studies showed a transient increase of BNP levels from baseline to discharge, followed by a stepwise decrease until 1 year. The authors related the transient increase in BNP to the transient left ventricle dysfunction with depression of both systolic and diastolic left ventricular (LV) function associated with TAVI (30).

In contrast, a more recent study indicated that a high preprocedural BNP, and a rise in BNP at 30 days independently predicted 1 year outcome post-TF or transaxillary TAVI (20). This result was confirmed in another study from the PARTNER trial (19) showing that an increase of BNP at 30 days was a predictor of 1 year mortality of transfemoral TAVI patients, as was moderate or severe aortic regurgitation over 1 year, and Society of Thoracic Surgeons (STS) score. Therefore, a rise in BNP at 30 days from baseline could provide prognostic information that should prompt careful clinical evaluation of these patients (Table 1).

Koskinas et al described an association between a high baseline BNP and a higher risk of all-cause death and cardiovascular death at 2 years, and a more frequent occurrence of VARC-2 clinical endpoints at 1 year (21). In this study, BNP levels increased or remained unchanged from baseline to discharge in 35% of patients, while these levels decreased in 65% of them. A baseline-to-discharge decrease was related to New York Heart Association functional improvement. Patients with persistently high BNP before intervention and at discharge had increased rates of death at 2 years. The same authors compared the prognostic values of BNP and NT-proBNP, revealing superiority of postprocedural NT-proBNP to BNP as a predictor of all-cause mortality at 2 years. Another study analyzed the prognostic value of preprocedural NT-proBNP ratio, defined as the ratio of measured NT-proBNP to maximal normal NT-proBNP values specific for age and gender, on short- and long-term mortality (31). The authors showed that baseline NT-proBNP ratio could predict all-cause mortality at 30 days and 1 year post-TAVI. Finally, in a later study, preinterventional levels of mid-regional (MR), pro-adrenomedullin (MR-proADM), and MR-pro-A-type natriuretic peptide (MR-proANP) and N-terminal pro-natriuretic peptide (NT-proBNP) were associated with 1 year cardiovascular events and all-cause mortality, while no association was found with 30 day outcome (32). Among most recently studied biomarkers, baseline levels of carbohydrate antigen 125 were reported to be superior to NT-proBNP to predict adverse outcome of TAVI (33).

Thus, altogether these studies depict some prognostic value of periprocedural BNP in TAVI that should be validated in larger multicenter studies in order to foster their implementation in current clinical practice.

### Markers of Inflammation and Myocardial Stress

#### GDF-15

A prospective observational study was conducted that compared the prognostic value of risk scores (logistic European System for Cardiac Operative Risk Evaluation [EuroSCORE], EuroSCORE II, Society of Thoracic Surgeons predicted risk of mortality, and German aortic valve score) and circulating biomarkers (high-sensitivity C-reactive protein [hsCRP], growth differentiation factor [GDF]-15, interleukin-6, interleukin-8, and NT-proBNP) to predict all-cause mortality and rehospitalization during the first year after TAVI (34). Strikingly, GDF-15, a cytokine belonging to the family of transforming growth factor-β, appeared to be the best predictor of poor outcome when added to the logistic EuroSCORE and EuroSCORE II.

These results are in agreement with another study in which high preintervention GDF-15 levels were associated with reduced time survival post-TAVI, and were superior to NT-proBNP for patient risk stratification (35). Interestingly, high GDF-15 levels were significantly associated with several variables of poor outcome, such as reduced kidney function, diabetes, STS score, high creatinine and NT-proBNP levels, and VARC-2 criteria, suggesting that GDF-15 could integrate numerous complicating factors that could contribute to poor TAVI outcome.

Among eight biomarkers measured prior to valve replacement (GDF-15, soluble ST2 [sST2], NT-proBNP, galectin-3 [GAL-3], hs-cTnT, myeloperoxidase, hsCRP, and monocyte chemotactic protein-1 [MCP-1]), Lindman et al identified a combination of elevated levels of GDF-15, sST2 and NT-proBNP as the best predictors of 1 year mortality post-TAVI (36). However, since this study included both TAVI and patients who underwent surgical valve replacement, the utility of these three biomarkers should still be evaluated in specific populations of TAVI patients.

A recent study assessed the association of preprocedural BNP, hs-TnI, CRP, GDF-15, GAL-3, and cystatin-C with LV mycordial recovery with long-term all-cause mortality. Again, GDF-15 was strongly associated with all-cause mortality, as was CRP. GDF-15 improved the risk model when added to the STS score. Though frailty has been associated with worse 1 year outcome post-TAVR, in this study, frailty alone was not superior to GDF-15 and did not significantly improve net reclassification when added to STS score. The authors also found that a lower baseline level of GDF-15 predicted improvement of global longitudinal strain (GLS) at 1 year follow-up, which may partly explain the effect on survival. Notably, GLS at baseline was not as strongly related to outcome as GDF-15 and CRP. GLS at 1 month could, however, predict 1 year mortality. In addition, this study uncovered an intriguing correlation between GDF-15 and left ventricular mass index.

Thus, baseline GDF-15 appears as a promising biomarker that could improve current risk prediction models for patients undergoing TAVI. Furthermore, these findings indicate that inflammation may play a major role in ventricular remodeling and recovery post-TAVI. Performing serial measurements of GDF-15 and CRP would thus be interesting to determine the effect of the TAVI procedure on the progression of the inflammatory process, and its impact on patient outcome.

GDF-15 has been associated with multiple cardiovascular outcomes, possibly due to its pleiotropic effects on inflammation, oxidative stress, endothelial dysfunction, myocardial stress, and aging. Of particular interest, several studies reported an association of GDF-15 with a risk of major bleeding in acute coronary syndrome patients on dual antiplatelet therapy (37, 38). However, no studies have evaluated the possible role of GDF-15 in TAVI-related bleeding events (see below), so far.

### Markers of Inflammation and Myocardial Stress

#### Soluble ST2

sST2 is an interleukin-1 receptor family member that acts as a decoy receptor for interleukin-33, and inhibits cardioprotective IL-33/ST2 signaling (39). Released following hemodynamic stress and cardiomyocyte strains (40), sST2 accurately predicts cardiovascular outcome of patients with acute and chronic heart failure. Consequently, sST2 was introduced in the ACC/AHA guidelines for risk stratification of patients (41). Our team showed an association of sST2 with outcome in aortic stenosis (42).

 sST2 levels increase during the 24 h following TAVI, probably related to periprocedular myocardial dysfunction (30). Three studies recently indicated that preprocedural soluble ST2 might have long-term prognostic value after TAVI. The first study showed an association of baseline sST2 with 1 year mortality, with no effect at 1 month (43). sST2 correlated significantly with echocardiographic parameters, CRP, creatinine, and BNP. In a second study, sST2 was independently associated with 1 year mortality after TAVI, as were logistic EuroSCORE, chronic renal failure, and left ventricular ejection fraction (44). However, it was not superior to NT-proBNP or surgical risk scores (STS-PROM) for risk assessment, possibly due to confounding effect of inflammation on sST2 levels. In a third study, sST2 predicted mortality and the occurrence of major cardiovascular events post-TAVI (45). In contrast to the study of Stundl et al, adding sST2 to the STS score improved risk prediction of 2 year mortality.

Again, regarding sST2, future larger studies are awaited to validate these findings.

### Markers of Hemostasis Imbalance

In aortic stenosis, high shear stress through aortic valve induces a loss of high molecular weight von Willebrand factor (vWF) multimers (HMWM), platelet activation and release of platelet granule content (46). Increased activation of coagulation with concurrent hypofibrinolysis is also observed (47), all this contributing to the dual clinical picture of AS, characterized by mild bleeding tendency (48), and high thrombotic risk.

Thromboembolic events, primarily stroke, are serious complications of TAVI procedures, occurring in up to 3–5% of patients. In addition, TAVI causes thrombocytopenia in one-third of patients. Importantly, while thrombocytopenia often resolves at discharge, persistent thrombocytopenia accurately predict 1 year mortality post-TAVI (49). Moreover, post-TAVI thrombocytopenia was found to be related to early post-procedural adverse events, including vascular complications, bleeding, and the need for multiple blood transfusions. To prevent TAVI-associated thromboembolic events and thrombocytopenia, a 3- to 6 month dual antiplatelet therapy (DAPT) is currently recommended for all approved balloon expandable and self-expandable transcatheter heart valve prostheses.

To determine which factors may explain the drop in platelet count that occurs after TAVI, Mitrosz et al (50) have prospectivelly analyzed changes in platelet count, along with markers of coagulation activation (F1 +2) and soluble markers of platelet activation (P-selectin, PF4) in a small cohort of severe AS, before TAVI and on the three postoperative days. While platelet reduction shortly after TAVI procedure was mostly influenced by the amount of contrast agent applied during the procedure, levels of PF4 and P-selectin positively correlated with the drop of platelet count, suggesting that thrombocytopenia is secondary to platelet activation. In-hospital major adverse cardiovascular events were observed more frequently in patients with more severe platelet count decrease (51). In another study, levels of thrombin-antithrombin complexes (TAT), plasmin-α₂-antiplasmin complex (PAP), and D-dimers significantly increased after TAVI, and D-dimer as well as PAP remained elevated until day 7, indicative of TAVI-induced increased thrombin formation and fibrinolysis (52). Post-TAVI thrombocytopenia occurred in one-fifth of patients and was associated with a significantly higher incidence of post-TAVI complications, e.g., acute kidney injury and vascular complications, whereas no impact of activated coagulation on thrombocytopenia was observed.

Thus, altogether these studies indicate that consumption of activated platelets might be the mechanism leading to thrombocytopenia after TAVI. Therefore, periprocedural platelet activation markers may potentially represent predictors of adverse outcome.

Bleeding is a more common complication of TAVI than thromboembolic events, as major and life-threatening bleeding (MLTB) according to VARC-2 can occur in up to 30% of patients (53, 54). Of note, periprocedural bleeding independently predicts all-cause mortality after TAVI (53). High mean platelet volume (MPV) and low platelet distribution width (PDW) were associated with increased risk of any bleeding and MLTB (55). Since larger platelets are more reactive and are believed to increase thromboembolic risk (56–58), this finding may be surprising. However, it is possible that high MPV could be a consequence of patient’s health state, making them more prone to bleeding. It has been shown that MPV progressively normalizes during the days following TAVI, in parallel with NT-proBNP and hemodynamic parameters (59), but its relation with patient outcome has not been investigated yet.

Importantly, a decrease of platelet reactivity is probably not the only determinant of bleeding post-TAVI. Acquired von Willebrand disease may also play a role. However, to date, evidence for a link between vWF deficiency and overt bleeding in TAVI is lacking. Indeed, the loss of HMWM does not always associate with bleeding events after valve replacement (48, 60). Though, it has recently been shown that recovery of HMWM levels post-TAVI could be used as a marker of postprocedural paravalvular regurgitation, with a positive effect on 1 year mortality (61).

Finally, a high on-treatment platelet reactivity (HTPR) to clopidogrel, due to impaired response to this antiplatelet medication, appears to be very frequent in TAVI patients (62, 63). Yet, no studies have evaluated the association of HTPR with post-TAVI outcomes. The ARTE randomized clinical trial showed a reduction of death, myocardial infarction, stroke, transient ischemic attack, or MLTB within the 3 months following TAVI with aspirin monotherapy versus DAPT (64). Thus, since there is currently no approved alternative to clopidogrel medication in >75 years TAVI patients (65), larger clinical trials aimed at defining the optimal antithrombotic regimen in these patients are awaited.

Strikingly, a recent study indicated that periprocedural changes in plasma markers of inflammation, interleukin-6 and S100A8/A9, could predict the decline in platelet count in the days following TAVI (66). A drop in platelet count and inhibition of agonist-induced platelet activation occurred in parallel with an increase of the inflammation markers following valve deployment. Thus, the inflammatory process elicited by TAVI may contribute to postprocedural thrombocytopenia. This is in line with a study showing that severe systemic inflammatory response syndrome (SIRS) was related to higher 6 month all-cause mortality after TAVI (67). This concept warrants further investigation.

## Conclusion

In conclusion, blood biomarkers may enrich current risk scores in the future. BNP is readily available and easy to perform. Large studies will clarify the role of further markers.

## Author Contributions

CO wrote the manuscript. PL, AN, and JB provided intellectual contributions and edited the manuscript.

## Conflict of Interest Statement

The authors declare that the research was conducted in the absence of any commercial or financial relationships that could be construed as a potential conflict of interest.
